# UVB-dependent inhibition of lipin-1 protects against proinflammatory responses in human keratinocytes

**DOI:** 10.1038/s12276-020-0388-y

**Published:** 2020-02-21

**Authors:** Minjung Chae, Eui Dong Son, Il-Hong Bae, Eun-Gyung Cho, Hyoung-June Kim, Ji-Yong Jung

**Affiliations:** Basic Research and Innovation Division, Bioscience Laboratory, AmorePacific Corporation R&D Center, Yongin-si, Gyeonggi-do South Korea

**Keywords:** Lipid signalling, Interleukins

## Abstract

Lipin-1 is an Mg^2+^-dependent phosphatidate phosphatase (PAP1) that catalyzes a critical step in the synthesis of glycerophospholipids and is also a cotranscriptional regulator. The role of lipin-1 in the regulation of inflammatory responses has been extensively studied in various cell types but not in skin cells. In the present study, the function of lipin-1 in UVB**-**induced proinflammatory responses was assessed in normal human epidermal keratinocytes (NHEKs). UVB radiation downregulated lipin-1 expression. Lipin-1 inhibition was mediated by UVB-dependent sterol-response element binding protein-1 (SREBP-1) inhibition. The UVB-dependent inhibition of lipin-1 and SREBP-1 was mediated by AMPK activation. UVB-induced activation of JNK was dependent on AMPK activation and mediated lipin-1 inhibition. Prevention of UVB**-**mediated lipin-1 repression by introducing a lipin-1 expression vector stimulated IL-6 and IL-8 production, suggesting that lipin-1 inhibition attenuates UVB-induced IL-6 and IL-8 production. The downregulation of lipin-1 ameliorated UVB-induced NF-ĸB phosphorylation, which might be attributed to the suppression of UVB-induced accumulation of free fatty acids (FFAs). Pharmacological inhibition of PAP1 with propranolol suppressed UVB-induced production of IL-6 and IL-8 in NHEKs and reconstituted human skin models. Taken together, lipin-1 is downregulated by exposure to UVB radiation, which confers protection against UVB-induced proinflammatory responses; therefore, the inhibition of lipin-1 is a potential strategy for photoaging.

## Skin health: An enzyme to target for UV protection

Reduced production and activity of an enzyme in skin cells helps protect them from damage caused by exposure to ultra-violet light. Minjung Chae and colleagues at the Amorepacific Corporation in Yongin, South Korea, identified an anti-inflammatory effect caused by the reduction in expression of the enzyme lipin-1 when skin cells are exposed to UVB radiation. These ultra-violet rays are associated with aging and increased risk of skin cancer. Lipin-1 is involved in making glycerophospholipid molecules, which are key components of the membranes surrounding and inside cells. Identifying the enzyme’s significance for inflammation in skin cells extends previous similar findings with other cell types. The research also uncovered aspects of the molecular mechanisms mediating the skin cell response. Inhibiting lipin-1 activity might reduce the damage sunlight causes to skin.

## Introduction

UV radiation (UVR) in sunlight is a principal environmental factor that contributes to the development of skin cancer, which is the most common malignancy worldwide. The wavelengths for UVB (280–315 nm) radiation are the most deleterious, as they can result in the development of skin cancer^1^. UVB radiation typically elicits acute inflammatory responses by stimulating the release of proinflammatory cytokines, including TNF-α, IL-1α, IL-6, and IL-8; this eventually leads to photoaging and skin cancer development^2,3^.

Lipin-1, encoded by the *Lpin1* gene, has dual functions in lipid synthesis and transcriptional regulation^4^. In the cytoplasm, lipin-1 acts as an Mg^2+^-dependent phosphatidic acid phosphatase (PAP1) that catalyzes the dephosphorylation of phosphatidic acid (PA) to diacylglycerol (DAG), which is a precursor of stored triglycerides (TAGs) and several phospholipids^5^. In addition, lipin-1 is translocated into the nucleus and interacts with transcriptional coactivators, such as the peroxisome proliferator-activated receptor alpha (PPARα) family, sterol-response element binding protein-1 (SREBP-1), and nuclear factor of activated T cells isoform c4 (NFATc4)^4,6^. The phosphorylation of lipin-1 by mechanistic target of rapamycin complex-1 (mTORC-1) causes lipin-1 to localize from the cytoplasm to the nucleus^7–9^. Additionally, Tip60-mediated lipin-1 acetylation influences lipin-1 localization^10^.

The role of lipin-1 in regulating inflammatory responses is unknown and controversial. Lipin-1 has shown anti-inflammatory properties. In adipocytes, lipin-1 represses proinflammatory cytokines by inhibiting the transcriptional activity of NFATc4^11^. When an ethanol diet is provided, lipin-1 inhibition stimulates monocyte chemoattractant protein-1 (MCP-1), which is a regulator of inflammation, in adipose tissues^12^. Hepatic lipin-1 ablation augments the ethanol-induced expression of hepatic proinflammatory cytokines, such as TNF-α, IL-1β, lipocalin-2 (Lcn-2), and serum amyloid A-1 (Saa-1), in mice^13^. Conversely, the results of other studies have also demonstrated that lipin-1 contributes to proinflammatory effects. Lipin-1 mediates the generation of proinflammatory cytokines in Toll-like-receptor (TLR)-stimulated macrophages^14^. The loss of lipin-1 in myeloid cells attenuates hepatic inflammation after ethanol administration^15^.

We previously demonstrated that lipin-1 expression is critical for keratinocyte differentiation, as it controls the DAG level, activating protein kinase C (PKC) activity, particularly the alpha isoform^16^. However, little is known about the role of lipin-1 in inflammatory responses in skin cells, even though lipin-1 has been implicated in inflammation in various other cell types. To determine the role of lipin-1 in generating inflammatory responses upon exposure to UVB radiation, we examined the effect of UVB radiation on the expression level of lipin-1 and UVB-induced signals that regulate lipin-1 expression in normal human epidermal keratinocytes (NHEKs). We also investigated the effect of lipin-1 expression on UVB-induced proinflammatory cytokines and lipid accumulation. Finally, the effect of pharmacological inhibition of PAP1 with propranolol was tested to examine UVB-induced inflammatory cytokines in NHEKs and reconstituted human skin models.

## Materials and methods

### Cell culture and chemicals

Normal human epidermal keratinocytes (NHEKs) obtained from the neonatal foreskin were purchased from Lonza (Basel, Switzerland) and cultured in keratinocyte growth medium (KBM gold) with BulletKit (Lonza) containing insulin, human epidermal growth factor, bovine pituitary extract, hydrocortisone, epinephrine, transferrin, and gentamicin/amphotericin B. The cells were serially passaged until 70–80% confluence was achieved, which was no more than three times. AMPK (compound C), ERK (PD98059), p38 MAPK (SB203580), JNK (SP600125), fatty acid synthase (C75), and lipin-1 (propranolol) inhibitors were obtained from Sigma (MO, USA). The expression vector for pcDNA3.1-Flag-SREBP-1a and -1c was a gift from Dr. Jae Bum Kim (Seoul National University).

### UVB light apparatus

We used a Biosun UV irradiation system with a lamp that produces wavelengths at approximately 280–320 nm (*λ*_max_: 312 nm) (Vilber Lourmat, Marnes-la-Vallee, France) to generate UVB radiation. The apparatus had a culture dish tray, and the temperature did not exceed 30 °C during exposure. Before UVB radiation, the cells were washed with 1 ml phosphate-buffered saline (PBS), and 0.5 ml fresh PBS was added to each well. The cells were irradiated at the desired intensity without the plastic dish lid. After UVB irradiation, the cells were incubated again in basal medium, and treatments were performed at various time points prior to the harvest step.

### Quantitative real-time RT-PCR (qRT-PCR)

Total RNA was isolated using TRIzol reagent (Invitrogen, CA, USA), according to the manufacturer’s instructions. The RNA concentration was determined spectrophotometrically, and the integrity of the RNA was assessed using a BioAnalyzer 2100 (Agilent Technologies, CA, USA). Two micrograms of RNA was reverse-transcribed into cDNA using SuperScript III reverse transcriptase (Invitrogen) and aliquots were stored at −20 °C. TaqMan RT-PCR technology (7500Fast, Applied Biosystems, CA, USA) was used to determine the expression levels of selected target genes. The process included a denaturing step performed at 95 °C for 10 min; 50 cycles were performed at 95 °C for 15 s and at 60 °C for 1 min. The following TaqMan probes were used for qRT-PCR analysis: lipin-1 (Hs00299515_m1), SREBP-1 (Hs01088691_m1), ACACA (Hs01046047_m1), ACLY (Hs00982738_m1), SCD1 (Hs01682761_m1), IL-6 (Hs00985639_m1), IL-8 (Hs00174103_m1), ATGL (Hs00386101_m1), and HSL (Hs00943410_m1). The probe for RPL13A (Hs04194366_g1) (Applied Biosystems) was also amplified to normalize the variations in cDNA levels across different samples. For total lipin-1, lipin-1α, and lipin-1β, primer sets were synthesized as described previously^17^, and mRNA levels were measured via SYBR GREEN (Applied Biosystems) RT-PCR.

### Western blot analysis

To prepare cell lysates, NHEKs were washed with ice-cold PBS and lysed in RIPA buffer (50 mM Tris-HCl pH 7.4, 150 mM NaCl, 0.5% sodium deoxycholate, 0.1% SDS, and 1% NP-40) in the presence of a protease and phosphatase inhibitor cocktail (Sigma). The lysates were then centrifuged at 15,000 × *g* for 20 min, and the supernatants were used for analysis. Protein concentrations were determined using a BCA kit (Sigma), using bovine serum albumin (BSA) as the standard. Equal amounts of protein (40 μg/well) from cell lysates were loaded and separated using 8–12% gradient SDS-PAGE and transferred onto PVDF membranes. The membranes were blocked in 3% BSA in TBST (20 mM Tris-HCl, pH 8.5, 150 mM NaCl, and 0.5% Tween) at room temperature for 30 min. The blots were incubated at 4 °C with anti-lipin-1 (R&D Systems, MN, USA), anti-SREBP-1, anti-GAPDH (Sigma), anti-phospho-JNK (T183/Y185), anti-JNK, anti-phospho-AMPK (Thr172), anti-AMPK, anti-phospho-NF-kB p65, and anti-NF-kB p65 (Cell Signaling, MA, USA) antibodies overnight in 3% BSA in TBST. Horseradish peroxidase-conjugated goat anti-rabbit or rabbit anti-goat IgG secondary antibodies were obtained from Bio-Rad (CA, USA). The membranes were washed three times for 15 min in TBST, followed by incubation with the appropriate horseradish peroxidase-conjugated goat anti-rabbit or rabbit anti-goat IgG secondary antibodies for 1 h at room temperature. The membranes were washed and visualized using enhanced chemiluminescent reagent (ECL) for immunofluorescence staining (Amersham Pharmacia Biotech, NJ, USA). Image analysis of the immunoblots was performed using ImageQuant TL software (GE Healthcare Life Sciences, PA, USA).

### Transfection

Predesigned ON-TARGET plus human small interfering RNAs (siRNAs) against lipin-1 (#L01742701) and AMPKα1/2 (#L005027/#L005361) and nontargeting pool siRNA (#D-001810-10) were purchased from Dharmacon (CO, USA). NHEKs were plated 24 h before transfection and transfected via lipofection using Lipofectamine RNAiMAX (Invitrogen) and OPTI-MEM (Invitrogen) with 25 nM siRNA for 6 h. The medium was then changed to KBM-gold medium, which contained all the appropriate supplements.

The lipin-1 expression vector was generated as described previously^16^. To achieve SREBP-1a, SREBP-1c, or lipin-1 overexpression, NHEKs were seeded 24 h before transfection and transfected with 1 μg/ml plasmid using the X-tremeGENE HP DNA transfection reagent (Roche, Mannheim, Germany), according to the manufacturer’s instructions. We used a 1.5:1 ratio per μl of the X-tremeGENE HP DNA transfection reagent to each μg of the pCMV6-AC-GFP vector as a transfection control.

### Cytokine array and ELISA

Media from NHEKs stimulated with UVB radiation were harvested and centrifuged for 15 min at 4 °C. The supernatants were freeze-dried and used for multiple cytokine measurements with a human cytokine array C3 (Raybiotech, GA, USA), according to the manufacturer’s instructions. The IL-6 and IL-8 levels were quantified using IL-6 and IL-8 ELISA kits, respectively, according to the manufacturer’s instructions (R&D Systems).

### Lipid analysis

TAG and free fatty acid levels were determined using reagents from Abcam, according to the manufacturer’s instructions. TAG and free fatty acid levels were normalized to the protein levels. PA levels were measured with a total phosphatidic acid fluorometric assay kit (Cayman Chemical, MI, USA), according to the manufacturer’s instructions. The PA level was normalized to the protein level.

### Phosphodiesterase activity assay

Cells were lysed in RIPA buffer with a protease and phosphatase inhibitor cocktail. The lysates were then centrifuged at 15,000 × *g* for 20 min, and the supernatants were desalted using Zeba spin desalting columns with a 7 kD molecular weight cut-off (Thermo Scientific, MA, USA). Desalted lysates were assayed for phosphodiesterase activity using a kit from Abcam, according to the manufacturer’s instructions.

### Immunohistochemistry analysis of a skin model

A reconstituted human epidermis model (EPI-200) was purchased from MatTek Corp. (MA, USA) and maintained according to the manufacturer’s instructions. After UVB radiation was applied to the skin model, treatment with propranolol or vehicle was administered for 24 h. Culture media were analyzed by ELISA kits for IL-6 or IL-8, and the reconstituted human skin was fixed in 10% neutral phosphate-buffered formalin and embedded in paraffin. Tissue sections with a thickness of 4 μm were incubated with anti-IL-6 and IL-8 antibodies (Abcam). Then, HRP-conjugated donkey anti-rabbit IgG (H + L) antibodies (Novus, CO, USA) were used as secondary antibodies. Immunoreactivity was visualized using 3,3′-diaminobenzidine as a chromogen. The results were analyzed under a light microscope (BX53, Olympus, Tokyo, Japan), and photomicrographs were taken using a cooling digital camera (DP72, Olympus).

### Statistical analysis

Statistical comparisons between two groups or within multiple groups were performed using Student’s *t* test or one-way ANOVA, respectively, followed by Tukey’s post hoc test. Measurements for at least three independent experiments were obtained, and values are expressed as the means ± SD.

## Results

### UVB inhibits lipin-1 expression in keratinocytes

**Fig. 1:**
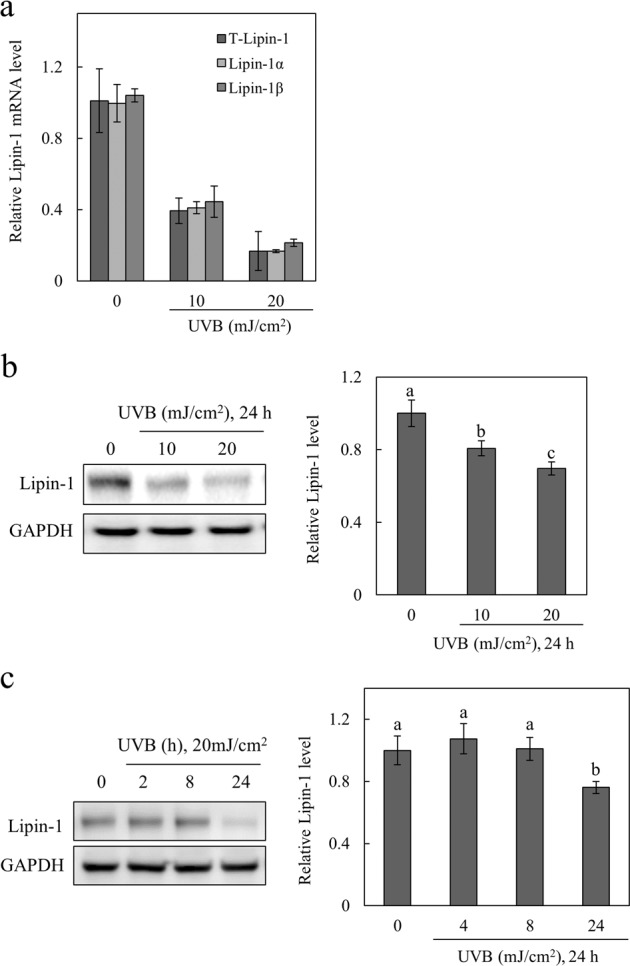
UVB downregulates lipin-1 expression in NHEKs. NHEKs were treated with different doses of UVB (0, 10, and 20 mJ/cm^2^) radiation and cultured for 24 h. **a** Total RNA was extracted from the cells, and relative mRNA levels of total (T) lipin-1, lipin-1α, and lipin-1β were measured via SYBR GREEN RT-PCR analysis. **b** Cell lysates were subjected to immunoblot analysis to assess lipin-1 expression levels. Protein levels of lipin-1 were normalized to GAPDH. **c** NHEKs were treated with 20 mJ/cm^2^ UVB radiation and harvested at the indicated time points (0, 4, 8, and 24 h), and cell lysates were immunoblotted with anti-lipin-1 antibody. Protein levels of lipin-1 were normalized to GAPDH. All data (mean ± SD) represent three independent experiments. Means without a common letter differ; *P* < 0.05.

To examine the effect of UV radiation on lipin-1 expression, the gene and protein expression levels of lipin-1 were measured after two different doses of UVB (10 and 20 mJ/cm^2^). Cell viability was not affected by these doses. We analyzed the transcript levels of lipin-1 using quantitative real-time PCR and found that UVB radiation decreased the mRNA expression of total lipin-1, lipin-1α, and lipin-1β in a dose-dependent manner (Fig. 1a). No changes were observed in the lipin-1α/lipin-1β ratio after the two different doses of UVB radiation (data not shown), which indicated that UVB radiation did not influence the alternative slicing of lipin-1 mRNA. Exposure to UVB radiation resulted in a dose-dependent decline in the protein expression levels of lipin-1 (Fig. 1b) after 24 h (Fig. 1c). These data suggest that UVB radiation inhibits lipin-1 expression in keratinocytes.

### UVB-induced downregulation of lipin-1 is mediated through SREBP-1 inhibition

**Fig. 2:**
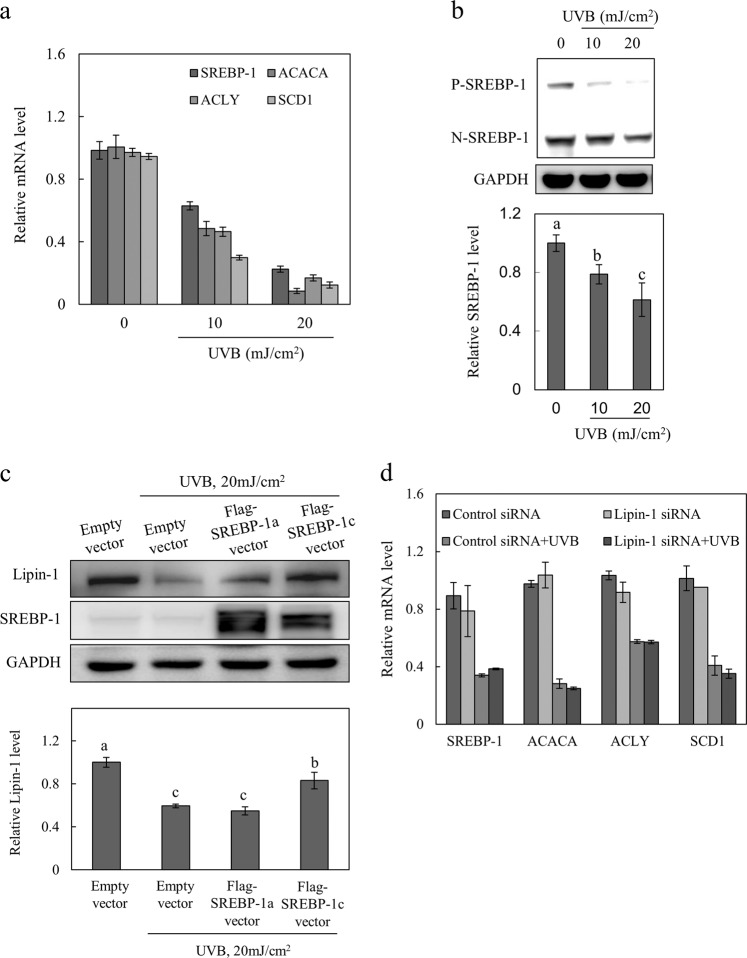
UVB-induced lipin-1 inhibition is mediated through SREBP-1 downregulation. NHEKs were treated with different doses of UVB (0, 10, and 20 mJ/cm^2^) and cultured for 24 h. **a** Total RNA was extracted from cells, and the relative mRNA levels of SREBP-1 and SREBP-1 target genes (ACACA, ACLY, and SCD1) were measured via qRT-PCR. **b** Cell lysates were immunoblotted to determine precursor (P) and nuclear (N) forms of SREBP-1. Protein levels of SREBP-1 were normalized to GAPDH. **c** NHEKs transfected overnight with the vector containing Flag-SREBP-1a, Flag-SREBP-1c cDNA, or empty pcDNA3.1 vector were exposed to 20 mJ/cm^2^ UVB radiation and cultured for 24 h. The protein levels of lipin-1 were normalized to GAPDH. All data (mean ± SD) represent three independent experiments. Means without a common letter differ; *P* < 0.05.

The expression of lipin-1 is regulated by SREBP-1, a major activator of hepatic lipogenesis that activates genes that are involved in fatty acid and triglyceride synthesis^18^. To investigate SREBP-1 signals that are produced after exposure to UVB radiation, we analyzed the transcript levels of SREBP-1 in UVB-stimulated keratinocytes via quantitative real-time PCR and found that SREBP-1 mRNA levels decreased in a dose-dependent manner (Fig. 2a). SREBP-1 target genes (ACACA, ACLY, and SCD1) were also downregulated by UVB radiation in a dose-dependent manner. There was a dose-dependent decrease in the levels of the precursor (P) and nuclear (N) forms of SREBP-1 (Fig. 2b). To explore the role of SREBP-1 signaling in UVB-mediated lipin-1 inhibition, Flag-SREBP-1a, Flag-SREBP-1c, or an empty vector was transfected into NHEKs that were then exposed to UVB radiation. The overexpression of Flag-SREBP-1c partially restored UVB-mediated lipin-1 inhibition (Fig. 2c), while siRNA-mediated lipin-1 inhibition did not affect SREBP-1 and SREBP-1 target genes; this indicated that SREBP-1 lies upstream of lipin-1 (Fig. 2d). Taken together, the UVB-dependent inhibition of lipin-1 is partly mediated through the downregulation of SREBP-1c.

### AMPK signaling cascade is responsible for UVB-dependent inhibition of lipin-1

**Fig. 3:**
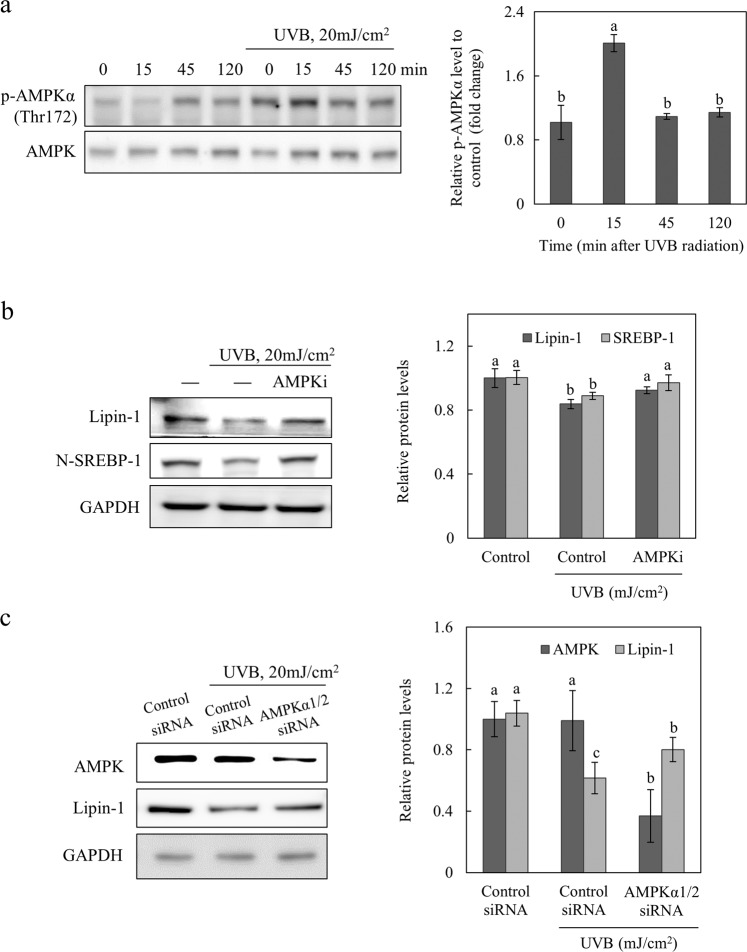
The AMPK signaling cascade is responsible for UVB-induced lipin-1 inhibition. **a** NHEKs exposed to 20 mJ/cm^2^ UVB were cultured for 0, 15, 45, and 120 min. The protein levels of p-AMPKα (Thr172) and AMPK were detected by western blotting. The levels of phosphorylated AMPKα (Thr172) were normalized to AMPK. Normalized p-AMPK levels relative to those of the control were analyzed at 0, 15, 45, and 120 min after UVB exposure. **b** NHEKs that were pretreated with the AMPK inhibitor (compound C, 10 μM) for 15 min were exposed to 20 mJ/cm^2^ UVB and incubated for 24 h. Lipin-1 or N-SREBP-1 levels were detected by western blotting and normalized to GAPDH. **c** NHEKs that were transfected with lipin-1 or control siRNA were exposed to 20 mJ/cm^2^ UVB and incubated for 24 h. AMPK or lipin-1 levels were detected by western blotting and normalized to GAPDH. All data (mean ± SD) represent three independent experiments. Means without a common letter differ; *P* < 0.05.

AMP-activated protein kinase (AMPK) is a serine/threonine kinase that senses the depletion of intracellular energy and stimulates catabolic pathways that generate ATP^19,20^. Ethanol-induced upregulation of lipin-1 mRNA expression is inhibited by AMPK activation (AICAR or overexpression of a constitutively active form of AMPK), and its levels recover with overexpression of the active nuclear form of SREBP-1 in hepatocytes^13^. To elucidate AMPK signaling in NHEKs stimulated by UVB radiation, AMPKα activity was assessed using phospho-AMPKα (Thr172) antibodies. Our results demonstrated that exposure of NHEKs to UV radiation transiently induced AMPKα phosphorylation 15 min after UVB exposure, and the levels were subsequently restored compared to those in control cells (Fig. 3a). To test the possible role of AMPK activation in lipin-1 expression, NHEKs were stimulated with UVB radiation and treated with the AMPK inhibitor compound C. After exposure to UVB radiation, cell viability was not affected by compound C (Supplementary Fig. 1). Our results showed that compound C restored lipin-1 downregulation after UVB exposure (Fig. 3b). Additionally, the UVB-mediated downregulation of the nuclear form of SREBP-1 recovered in the presence of compound C. To confirm that UVB-induced AMPK activation inhibited lipin-1 expression, AMPKα1/2 or control siRNA was transfected into NHEKs, and the cells were exposed to UVB radiation. The inhibition of AMPKα expression by AMPKα1/2 siRNA partially prevented the UVB-mediated inhibition of lipin-1 expression (Fig. 3c). Collectively, our data confirmed that UVB radiation activates AMPK, which might be responsible for lipin-1 inhibition.

### UVB-induced activation of JNK depends on the activation of AMPK and mediates lipin-1 inhibition

**Fig. 4:**
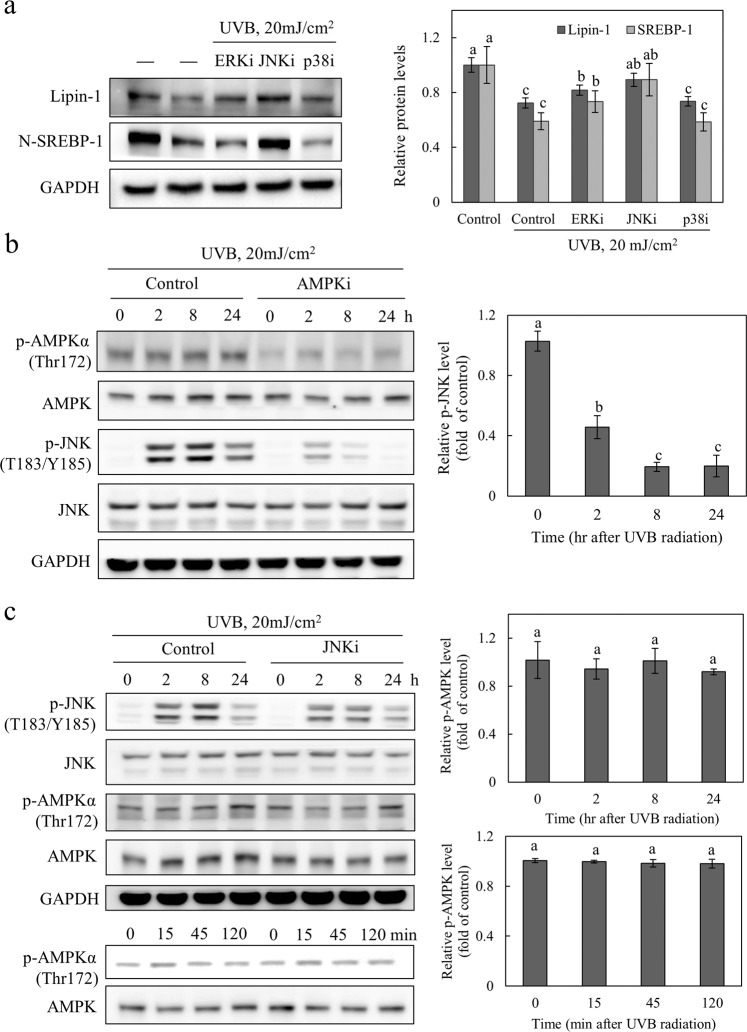
UVB-induced JNK activation depends on AMPK activation and mediates lipin-1 inhibition. **a** NHEKs were pretreated with the ERK (PD98059, 25 μM), p38 MAPK (SB203580, 1 μM), or JNK (SP600125, 5 μM) inhibitor for 15 min, followed by exposure to 20 mJ/cm^2^ UVB, and incubated for 24 h. Lipin-1 or N-SREBP-1 levels were detected by western blotting and normalized to GAPDH. **b** NHEKs were pretreated with the AMPK inhibitor (compound C, 10 μM) for 15 min, exposed to 20 mJ/cm^2^ UVB, and cultured for 0, 2, 8, and 24 h. p-AMPK (Thr172), AMPK, p-JNK (T132/Y185), and JNK levels were detected by western blotting. The levels of phosphorylated JNK (T132/Y185) were normalized to JNK, and the expression of normalized p-JNK relative to that of the control was analyzed at 0, 2, 8, and 24 h after exposure to UVB. **c** NHEKs that were pretreated with the JNK inhibitor (SP600125, 5 μM) for 15 min were exposed to 20 mJ/cm^2^ UVB and cultured for 0, 15, 45, and 120 min or 0, 2, 8, and 24 h. p-JNK (T132/Y185), JNK, p-AMPK (Thr172), and AMPK levels were detected by western blotting. The levels of phosphorylated AMPK (Thr172) were normalized to AMPK, and the expression of normalized p-AMPK relative to that of the control was analyzed at 0, 2, 8, and 24 h or 0, 15, 45, and 120 min after exposure to UVB. All data (mean ± SD) represent three independent experiments. Means without a common letter differ; *P* < 0.05.

The UVB-induced activation of MAP kinase has been extensively studied^21,22^. To elucidate the possible role of MAPK signaling pathways in UVB-dependent lipin-1 inhibition, NHEKs were stimulated with UVB and treated with kinase inhibitors, such as PD98059 (ERK inhibitor), SP600125 (JNK inhibitor), or SB203580 (p38 MAPK inhibitor). After exposure to UVB radiation, cell viability was not affected by kinase inhibitors (Supplementary Fig. 2). Our results showed that lipin-1 downregulation was restored after UVB exposure in the presence of SP600125 and, to a lesser extent, PD98059 (Fig. 4a). Additionally, JNK inhibition using SP600125 prevented the UVB-mediated downregulation of the nuclear forms of SREBP-1. These results indicate that the JNK-induced signaling pathway mediates lipin-1 downregulation, which is mediated by UVB radiation. The relationship between the UVB-induced activation of AMPK and JNK was investigated using AMPK or JNK inhibitors. In the presence of compound C, JNK phosphorylation was significantly reduced 2 h after UVB exposure, and this decrease was more pronounced after 8 h (Fig. 4b). However, after UVB exposure, AMPK phosphorylation was not affected for 2 or 24 h in the presence of the JNK inhibitor (Fig. 4c). These results indicate that UVB-induced AMPK signaling occurs upstream of JNK activation. Taken together, the UVB-induced activation of JNK, which is dependent on AMPK activation, contributes to lipin-1 inhibition.

### Lipin-1 regulates UVB-induced IL-6 and IL-8 production

**Fig. 5:**
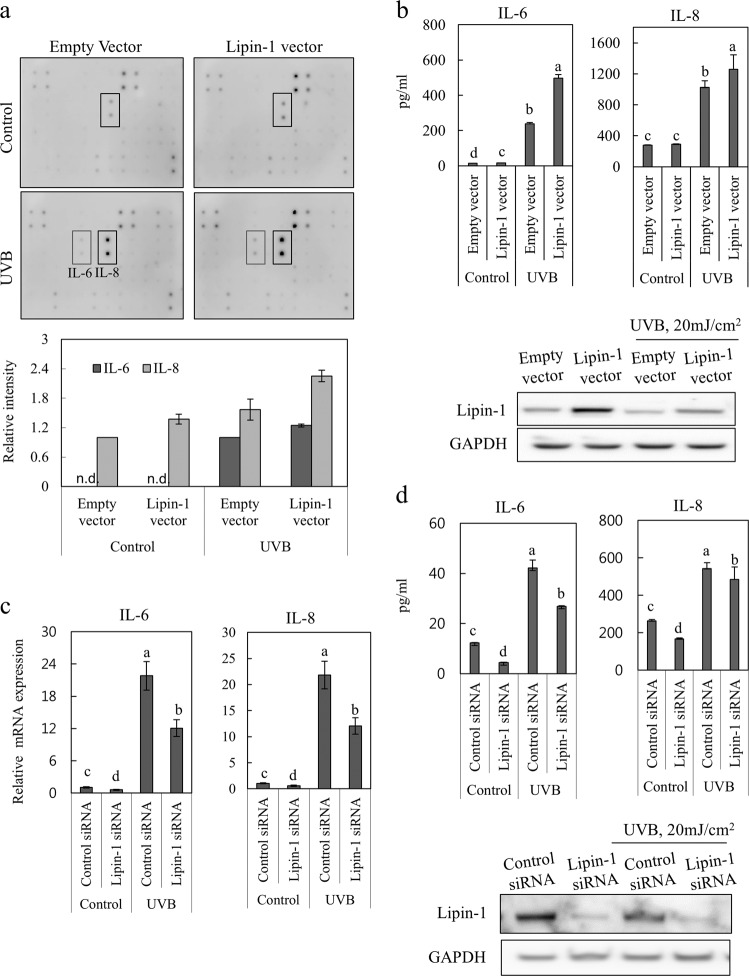
UVB-dependent lipin-1 downregulation attenuates UVB-induced IL-6 and IL-8 production. NHEKs that were transfected with the vector containing lipin-1 cDNA or pCMV-AC empty vector were exposed to 20 mJ/cm^2^ UVB and cultured for 24 h. **a** Culture media were harvested for cytokine array analysis. Representative altered spots were analyzed via densitometry. The data are presented as the mean ± SD values of the intensities of spots in two independent experiments. **b** Cell lysates were subjected to immunoblot analysis to confirm lipin-1 overexpression. Secreted IL-6 and IL-8 were detected in culture supernatants using specific ELISA kits. **c** NHEKs that were transfected with lipin-1 or control siRNA for 24 h were exposed to 20 mJ/cm^2^ UVB and cultured for 24 h. Total RNA was extracted from cells, and relative mRNA levels of IL-6 and IL-8 were measured via qRT-PCR. **d** Cell lysates were subjected to immunoblot analysis to confirm lipin-1 knockdown. The culture media were harvested, and IL-6 and IL-8 levels were detected via specific ELISA kits. **b**–**d** The data (mean ± SD) represent three independent experiments. Means without a common letter differ; *P* < 0.05.

Upon exposure to UVB radiation in the epidermis, several types of cells, especially keratinocytes, produce proinflammatory cytokines, such as IL-1α, IL-6, IL-8 and tumor necrosis factor (TNF)-α, leading to skin inflammation^23–25^. To determine whether lipin-1 is involved in the generation of proinflammatory responses to UVB radiation, NHEKs were transfected with vectors containing lipin-1 cDNA or the pCMV-AC empty vector as a negative control, stimulated with UVB radiation and analyzed to determine changes in the levels of proinflammatory factors using the Raybio human cytokine assay. Evaluation of the expression of proinflammatory proteins indicated that UVB-induced IL-6 and IL-8 production was increased by the overexpression of lipin-1 (Fig. 5a). To confirm the involvement of lipin-1 expression in IL-6 and IL-8 production, the levels of these proteins were determined in the supernatants of lipin-1-overexpressed or control NHEKs after UVB exposure. We measured the increased secretion of IL-6 and IL-8 in NHEKs that were transfected with the lipin-1 vector after UVB stimulation (Fig. 5b). To confirm the involvement of lipin-1 expression in IL-6 and IL-8 production, the mRNA levels of these factors were analyzed in NHEKs that were transfected with lipin-1 or control siRNA. Lipin-1 knockdown (KD) in cells that were exposed to UVB radiation resulted in a reduction in the mRNA levels of IL-6 and IL-8 (Fig. 5c). We analyzed the supernatants of UVB-stimulated NHEKs and found that there was a reduction in IL-6 and IL-8 secretion in lipin-1 KD cells (Fig. 5d). Taken together, these results suggest that UVB-mediated downregulation of lipin-1 contributes to attenuating IL-6 and IL-8 synthesis in NHEKs.

### Lipin-1 downregulation inhibits UVB-induced NF-ĸB phosphorylation and free fatty acid accumulation

**Fig. 6:**
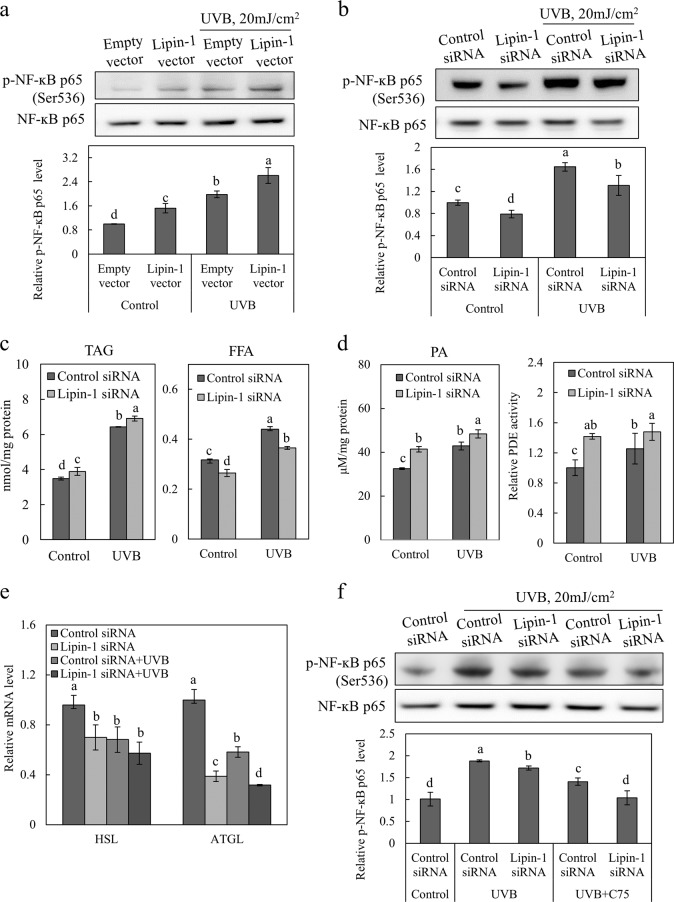
Lipin-1 regulates UVB-induced NF-ĸB phosphorylation, which might be mediated by FFA levels. **a** NHEKs that were transfected with the vector containing lipin-1 cDNA or pCMV-AC empty vector were exposed to 20 mJ/cm^2^ UVB and cultured for 24 h. Immunoblot analysis was used to determine p-NF-ĸB p65 (Ser536) and NF-ĸB p65 expression levels in cell lysates. The levels of phosphorylated NF-ĸB p65 (Ser536) were normalized to p65 NF-ĸB. **b** NHEKs that were transfected with lipin-1 or control siRNA were exposed to 20 mJ/cm^2^ UVB and cultured for 24 h. Immunoblot analysis was used to determine p-NF-ĸB p65 (Ser536) and NF-ĸB p65 expression levels in cell lysates. The levels of phosphorylated NF-ĸB p65 (Ser536) were normalized to p65 NF-ĸB. **c**, **d** Total lipids were extracted from cells, and biochemical analysis was used to determine the TAG, FFA and PA levels. PDE activity was assayed in cell lysates. **e** Total RNA was extracted, and the relative mRNA levels of ATGL and HSL were measured via qRT-PCR. **f** NHEKs were transfected with lipin-1 siRNA or control siRNA for 24 h, exposed to 20 mJ/cm^2^ UVB radiation, and incubated in the presence of C75 (5 μM) for 24 h. Immunoblot analysis was used to determine p-NF-ĸB p65 (Ser536) and NF-ĸB p65 expression levels in cell lysates. The levels of phosphorylated NF-ĸB p65 (Ser536) were normalized to NF-ĸB p65. All data (mean ± SD) represent three independent experiments. Means without a common letter differ; *P* < 0.05.

Because UVB-induced activation of the NF-ĸB signaling pathway is a well-characterized regulator of cytokines, we tested the effect of lipin-1 overexpression or knockdown on NF-ĸB activation after UVB exposure. NF-ĸB activity was assessed using phospho-p65 NF-ĸB (Ser536) antibodies. Upon UVB exposure, cells in which lipin-1 was overexpressed or knocked down exhibited augmented (Fig. 6a) or reduced (Fig. 6b) phosphorylation of NF-ĸB, respectively. Taken together, these results suggest that lipin-1 regulates UVB-induced activation of NF-ĸB in keratinocytes.

Because lipin-1-produced DAG may serve as a TAG precursor, we evaluated the effect of lipin-1 on the production of UVB-mediated TAG and FFA levels, as detected by biochemical analysis. UVB radiation promoted TAG and FFA synthesis in NHEKs (Fig. 6c). Interestingly, lipin-1 KD increased TAG levels but reduced FFA levels in both UVB-treated and control cells. To analyze the unexpected lipid content of lipin-1 KD cells, we measured phosphatidic acid levels because the role of phosphatidic acid as a second messenger has been linked to the regulation of PDE4 activity^26,27^. Phosphodiesterases (PDEs) are enzymes that catalyze the hydrolysis of cAMP and terminate the lipolytic activation of cAMP^28^. We observed that UVB radiation and lipin-1 knockdown promoted PA levels and PDE activity (Fig. 6d). We examined the effects of lipin-1 KD on the expression of genes related to TAG lipolysis that lead to the breakdown of TAGs into FFAs and glycerol. TG lipase (ATGL) and hormone-sensitive lipase (HSL) levels were reduced in lipin-1 KD cells (Fig. 6e), indicating that lipin-1 is involved in TAG lipolysis but not TAG synthesis in keratinocytes. UVB radiation also inhibited the expression of ATGL and HSL, which are linked to UVB-induced TAG accumulation. Taken together, these results suggest that lipin-1 downregulation contributes to UVB-induced TAG accumulation through inhibition of TAG lipolysis.

Free fatty acids induce skin inflammation^29,30^. Lipin-1 KD caused the FFA content to decrease compared to that in both UVB-treated and control cells, which might be linked to the inhibition of TAG lipolysis. To confirm the effect of FFA accumulation on the UVB-induced activation of NF-ĸB, we treated cells with C75 (FAS inhibitor) and examined NF-ĸB phosphorylation levels after UVB exposure in lipin-1 KD and control cells. After exposure to UVB radiation, cell viability was not affected by C75 (Supplementary Fig. 3a). C75 treatment inhibited the phosphorylation of NF-ĸB in lipin-1- or control siRNA-transfected cells (Fig. 6f). Additionally, IL-6 and IL-8 levels were drastically inhibited by C75 treatment (Supplementary Fig. 3b, c). These data suggest that the inhibition of UVB-induced FFA accumulation inhibits NF-ĸB activation, thereby attenuating the production of IL-6 and IL-8. Therefore, a reduction in FFA levels by lipin-1 siRNA might be attributed to the reduction in the levels of NF-ĸB phosphorylation induced by UVB exposure in NHEKs. Collectively, lipin-1 downregulation attenuates UVB-induced FFA accumulation, which might alleviate UVB-induced NF-ĸB activation.

### Propranolol abrogates UVB-induced production of IL-6 and IL-8

**Fig. 7:**
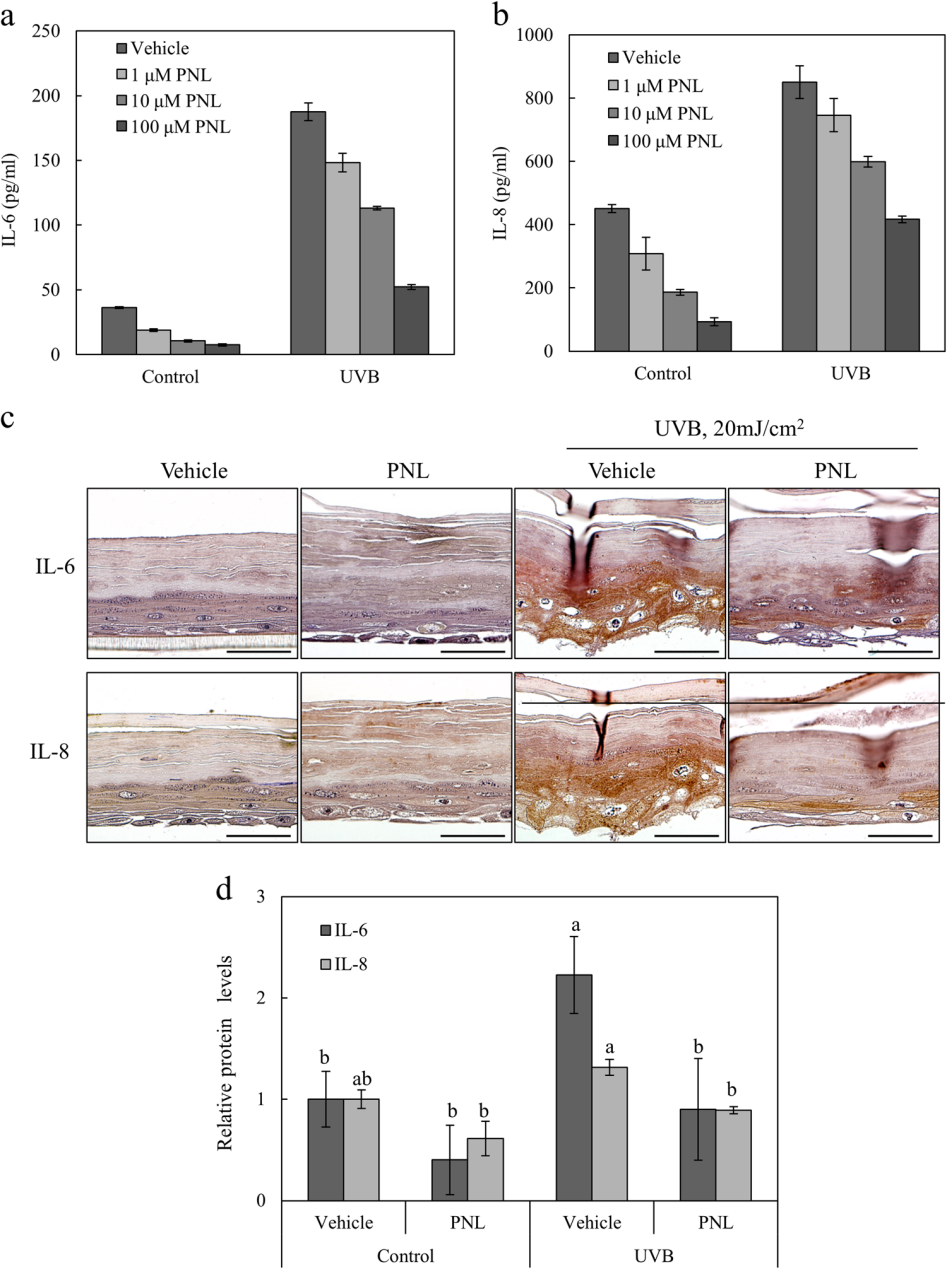
Propranolol sensitizes UVB-induced IL-6 and IL-8 production. NHEKs that were exposed to 20 mJ/cm^2^ UVB were treated with different concentrations of propranolol (PNL; 0, 1, 10, and 100 μM) for 24 h. **a**, **b** Secreted IL-6 and IL-8 were detected in culture supernatants using specific ELISA kits. All data (mean ± SD) represent three independent experiments. **c** A reconstituted human skin model was stimulated with 20 mJ/cm^2^ UVB, treated with 10 μM propranolol, and fixed at 24 h for immunohistochemical analyses. IL-6 and IL-8 levels were determined using each antibody. Scale bars: 50 μm. **d** Culture media were analyzed by ELISA kits for IL-6 or IL-8. All data (mean ± SD) represent three independent experiments. Means without a common letter differ; *P* < 0.05.

Propranolol is an inhibitor of PAP1 activity^31,32^. We determined the effect of the pharmacological inhibition of PAP1 on IL-6 and IL-8 production in both UVB-irradiated and control NHEKs after UVB exposure. Propranolol at the indicated concentrations did not affect cell viability (Supplementary Fig. 4a). Propranolol inhibited IL-6 and IL-8 production in a dose-dependent manner (Fig. 7a, b). We determined the effect of propranolol on IL-6 and IL-8 production in both UVB-irradiated and control NHEKs after UVB exposure. Propranolol reduced the phosphorylation of NF-ĸB (Supplementary Fig. 4b). Pharmacological inhibition confirmed its role in the regulation of IL-6 and IL-8 levels. The anti-inflammatory properties of propranolol might be attributed to the suppression of NF-ĸB activity.

A reconstituted human skin model was treated with propranolol or vehicle after UVB exposure, and the IL-6 and IL-8 expression levels were determined. Immunohistochemical staining indicated that IL-6 and IL-8 levels were drastically increased in the UVB-treated model, and the increase was inhibited by propranolol (Fig. 7c). We also analyzed the supernatants of the UVB-stimulated skin model and found that there was a reduction in IL-6 and IL-8 secretion levels in the propranolol-treated skin model (Fig. 7d); these results were consistent with those of previous studies. These results suggest that UVB-induced IL-6 and IL-8 expression was suppressed by lipin-1/PAP1 inhibition in the epidermis.

## Discussion

**Fig. 8:**
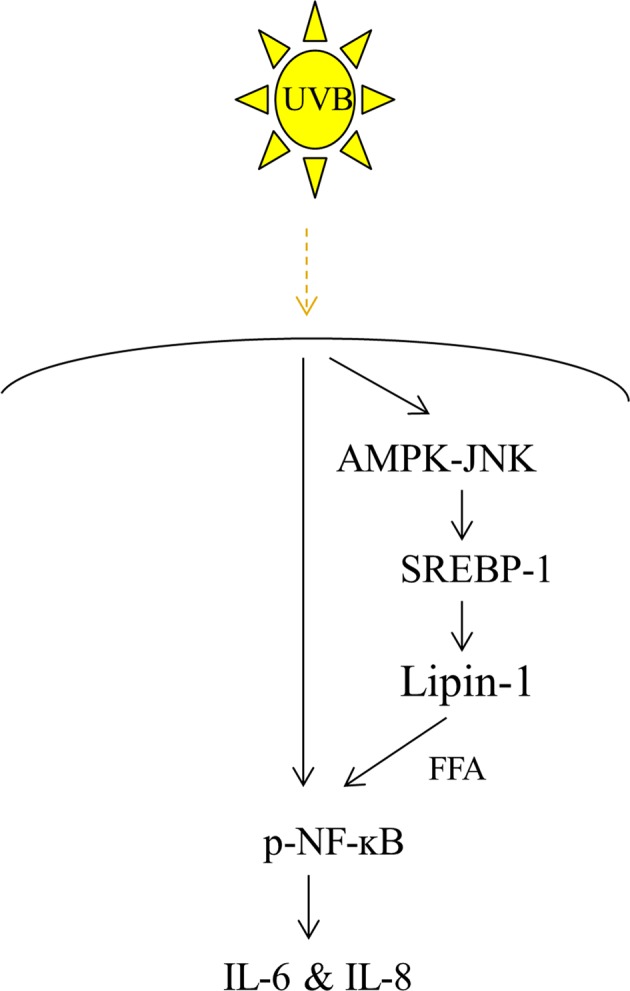
Proposed mechanism of the protective action of lipin-1 inhibition against UVB-induced proinflammatory responses. UVB radiation inhibits lipin-1 expression in NHEKs. This inhibition is mediated by the AMPK-JNK-SREBP-1 signaling axis. Lipin-1 inhibition suppresses UVB-induced IL-6 and IL-8 production. The anti-inflammatory effect of lipin-1 repression may be attributed to the suppression of NF-ĸB signaling and FFA accumulation.

The skin is the outermost barrier that protects organisms from pathogens and chemical or physical damage. UVR is the main causative agent of DNA mutations and altered gene expression patterns. The results of an oligonucleotide microarray analysis revealed that UVR treatment upregulated inflammation- and stress-related genes but downregulated metabolism- and adhesion-related transcript levels in human keratinocytes^33^. This modification acts as a self-protection mechanism against stress. However, very little is known about the roles of these transcript modifications. In the present study, we investigated the effect of UVB radiation on lipin-1 expression in NHEKs and the underlying mechanism and determined the role of lipin-1 expression in UVB-induced proinflammatory cytokines. Our results demonstrated that UVB radiation suppresses lipin-1 expression (Fig. 8); this inhibition is mediated by the AMPK/JNK/SREBP-1 signaling axis in NHEKs. Preventing the UVB-induced decline in lipin-1 levels stimulated IL-6 and IL-8 production, indicating that UVB mediates the downregulation of lipin-1, and this attenuates IL-6 and IL-8 production. The role of lipin-1 in the regulation of IL-6 and IL-8 is mediated by NF-ĸB signaling; UVB-induced activation of NF-ĸB was relieved by inhibition of FFA accumulation. Our findings provide valuable insight into potential therapies for skin inflammation.

AMPK is a stress-activated kinase that has cell protective effects against a number of stimuli, such as hyperglycemia^34,35^, fatty acid palmitate^36^, TNF^36,37^, and hypoxia^38^. Although the effect of UV radiation on AMPK activation in the epidermis is controversial^39,40^, our results indicated that AMPK activation is induced temporarily after UV exposure. Inhibition of AMPK activation by an AMPK inhibitor (compound C) or siRNA rescued lipin-1 expression levels that were downregulated by UVB exposure. AMPK activation using metformin, a known AMPK activator, inhibits IL-6, TNF-α, and VEGF expression in HaCaT cells^41^. We hypothesized that the UVB-induced activation of AMPK would result in anti-inflammatory signaling via lipin-1 inhibition in keratinocytes. However, the detailed mechanism by which UVB radiation activates AMPK and the possible role of AMPK in UV-induced signal transduction still needs to be elucidated. Sterol regulatory elements (SREs), which are present within the human *lipin1* promoter region, are the preferred SREBP-1 binding sites^18^. Activation of SREBP-1 by ethanol mediates the upregulation of lipin-1 in hepatic cells^42^. To examine the possible involvement of AMPK-SREBP-1 signaling in UVB-mediated lipin-1 inhibition, we transfected the SREBP-1 vector into NHEKs or treated them with the AMPK inhibitor compound C. Similarly, UVB-induced lipin-1 inhibition was rescued by overexpression of SREBP-1c; the AMPK inhibitor compound C recovered not only lipin-1 but also SREBP-1 expression.

UV radiation promotes ERK, JNK, and p38 mitogen-activated protein kinase (MAPK) activation^21,22^. Therefore, we investigated whether UVB-induced lipin-1 downregulation was mediated by MAPK signaling. Interestingly, JNK inhibition restored both lipin-1 and SREBP-1 expression after UVB radiation. The inhibition of JNK by SP600125 induces the enzymes involved in lipid and steroid metabolism and regulates inositol phosphate and diacylglycerol signaling in keratinocytes^43^. In the epidermis, UV-induced activation of JNK is known to elicit proinflammatory signaling. However, this study indicated that UVB-induced JNK activation was responsible for lipin-1 inhibition, which induces anti-inflammatory effects in keratinocytes. These data suggest that JNK indirectly induces anti-inflammatory effects via the regulation of lipid metabolism. The contradictory functions of JNK might be attributable to the diversity of upstream and downstream JNK signaling. Inhibition of lipin-1 expression by the transfected lipin-1 vector was observed after UVB exposure, suggesting a broad inhibition of protein synthesis. ER stress leads to the general shutdown of transcription by phosphorylating eIF2α, which is mediated by PERK, a regulator of apoptosis^44^. UVB radiation is known to generate ER stress and unfolded protein responses in HaCaT keratinocytes^45^. Our data also indicated that PERK was increased by UVB radiation (Supplementary Fig. 5). ER signaling in UVB-irradiated keratinocytes may contribute to global inhibition of translation.

Excessive exposure to UVB radiation typically leads to skin disorders by stimulating inflammatory factors, such as IL-1, IL-6, IL-7, IL-8, IL-12, IL-15, TNF-α, monocyte chemoattractant protein (MCP)-1, and granulocyte-macrophage colony-stimulating factor (GM-CSF), in keratinocytes in the skin^24,25^. These cytokines are associated with the development of various inflammatory skin diseases, such as psoriasis and atopic dermatitis^46–48^. Among these cytokines, IL-6 and IL-8 are regulated by lipin-1, an enzyme that is involved in lipid metabolism. The effects of altered lipid metabolism induced by lipin-1 deficiency on the production of proinflammatory cytokines were investigated. There was a significant increase in the TAG levels in cells that were exposed to UVB radiation. Moreover, UVB-induced TAG was upregulated by lipin-1 knockdown, indicating that lipin-1 downregulation is responsible for UVB-mediated TAG accumulation. Lipin-1 controls the opposing pathways for TAG synthesis and degradation in adipocytes^27^. TAG accumulation was also previously observed in HaCaT cells at 24 h after narrow band (NB) UVB exposure, and these results were similar to those of our study^49^. The effect of TAG on cell survival is somewhat controversial. TAG accumulation protects nonadipose cells from apoptosis by incorporating free fatty acids into TAGs^50^. However, TAG accumulation facilitates cell death by inducing ceramide synthesis and reactive oxygen species production^51,52^. The role of TAG accumulation in the epidermis remains unclear. UVB also stimulated FFA accumulation, but the increase in the FFA level was less pronounced than that of TAG. UVB-induced FFA accumulation was inhibited by lipin-1 knockdown. The altered TAG and FFA levels in NHEKs that were transfected with lipin-1 siRNA might be due to the reduced expression of adipose tissue TG lipase (ATGL). Defective epidermal lipolysis leads to impaired TG turnover and FA release from cellular lipid droplets. ATGL knockout mice show TAG accumulation in the epidermis^53^. However, the mechanism by which lipin-1 regulates ATGL and HSL expression and the biological function of these effects need to be further investigated.

We found elevated levels of TAG and FFA in NHEKs that were exposed to UVB radiation and decreased expression of TAG- and FFA-related genes. These findings suggest that UVB exposure increases TAG and FFA levels, which in turn suppress the expression of TAG and FFA biosynthesis genes at both the transcriptional and translational levels via a feedback mechanism. In a previous study, IL-17A increased cellular cholesterol levels, which in turn resulted in the suppression of genes related to cholesterol and fatty acid biosynthesis in NHEKs^54^; these results are similar to those of our study. The expression of lipin-1, one of the products expressed by TAG and FFA biosynthesis genes, was impaired by UVB radiation, which attenuated proinflammatory cytokine levels. The intracellular accumulation of FFAs in cell leads to activation of proinflammatory pathways^29^. An influx of excess FFAs into keratinocytes by palmitic acid supplementation stimulates proinflammatory cytokines such as IL-6, IL-1β, and TNF-α via the activation of NF-ĸB^30^. The inhibition of FFA accumulation by C75 prevented the UVB-induced activation of NF-ĸB, indicating that FFA accumulation is responsible for UVB-induced NF-ĸB activation. Therefore, lipin-1-mediated FFA accumulation might regulate NF-ĸB activation in keratinocytes. Thus, the molecular mechanisms by which FFA regulates the phosphorylation of p65^ser536^ need to be investigated further. Lipin-1 activity is the first step in the synthesis of not only TAG but also some phospholipids, phosphatidylcholine (PC), and phosphatidylethanolamine (PE). PC has an important role in proinflammatory signaling pathways^55^. Thus, additional studies to determine the association between lipin-1-mediated phospholipid synthesis and proinflammatory signaling need to be performed.

Because our data demonstrated that lipin-1 downregulation has an anti-inflammatory effect in the epidermis, we then verified whether the pharmacological inhibition of PAP1 activity had therapeutic effects on UVB-mediated inflammation. Propranolol displays anti-inflammatory activity in cirrhosis^56^ and murine temporomandibular joint^57^. Propranolol inhibited UVB-induced proinflammatory responses in the epidermis. Thus, our data suggest that lipin-1 inhibition is a novel target for the prevention of UVB radiation-induced production of proinflammatory cytokines that lead to photoaging and skin cancer.

## Supplementary information

Supplementary data
