# Supplementary material for: UVB-dependent inhibition of lipin-1 protects against proinflammatory responses in human keratinocytes
Source: Exp Mol Med. 2020 Feb 21;52(2):293–307. doi: 10.1038/s12276-020-0388-y (PMC7062881; doi:10.1038/s12276-020-0388-y)
Supplement: Supplementary file 1 — Supplementary data [file 12276_2020_388_MOESM1_ESM.docx]

**Supplementary Material**

**UVB-dependent inhibition of lipin-1 protects against proinflammatory responses in human keratinocytes**

Minjung Chae*, Eui Dong Son, Il-Hong Bae, Eun-Gyung Cho, Hyoung-June Kim, Ji-Yong Jung

Basic Research and Innovation Division, Bioscience Laboratory, Amorepacific Corporation R&D Center, Yongin-si, Gyeonggi-do, South Korea

***Corresponding author**:

Minjung Chae, Ph.D.

Basic Research and Innovation Division, R & D Unit, AmorePacific Corp., 1920 Yonggu-daero, Giheung-gu, Yongin-si, Gyeonggi-do, South Korea

Tel: 82-31-280-2694, Fax: 82-31-899-2595

E-mail: [minjungc@amorepacific.com](mailto:minjungc@amorepacific.com)

**Supplementary Figure**

**
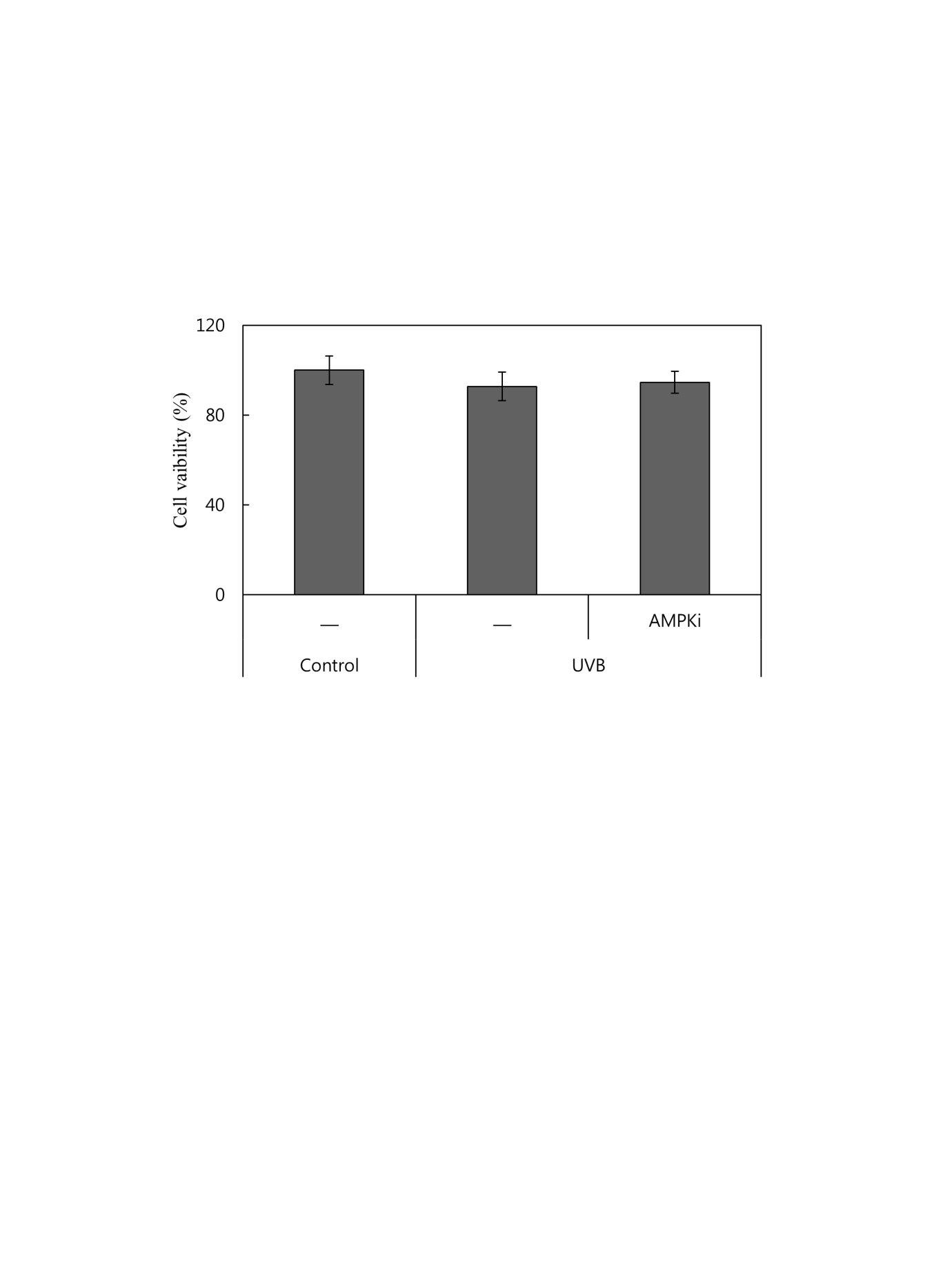
**

**Fig. S1. AMPK inhibitor does not affect cell viability after UVB exposure**

NHEKs pretreated with the AMPK inhibitor (compound C, 10 μM) for 15 min were exposed to 20 mJ/cm^2^ UVB and incubated for 24 h. Cell viability was determined by CCK-8 assay.

**
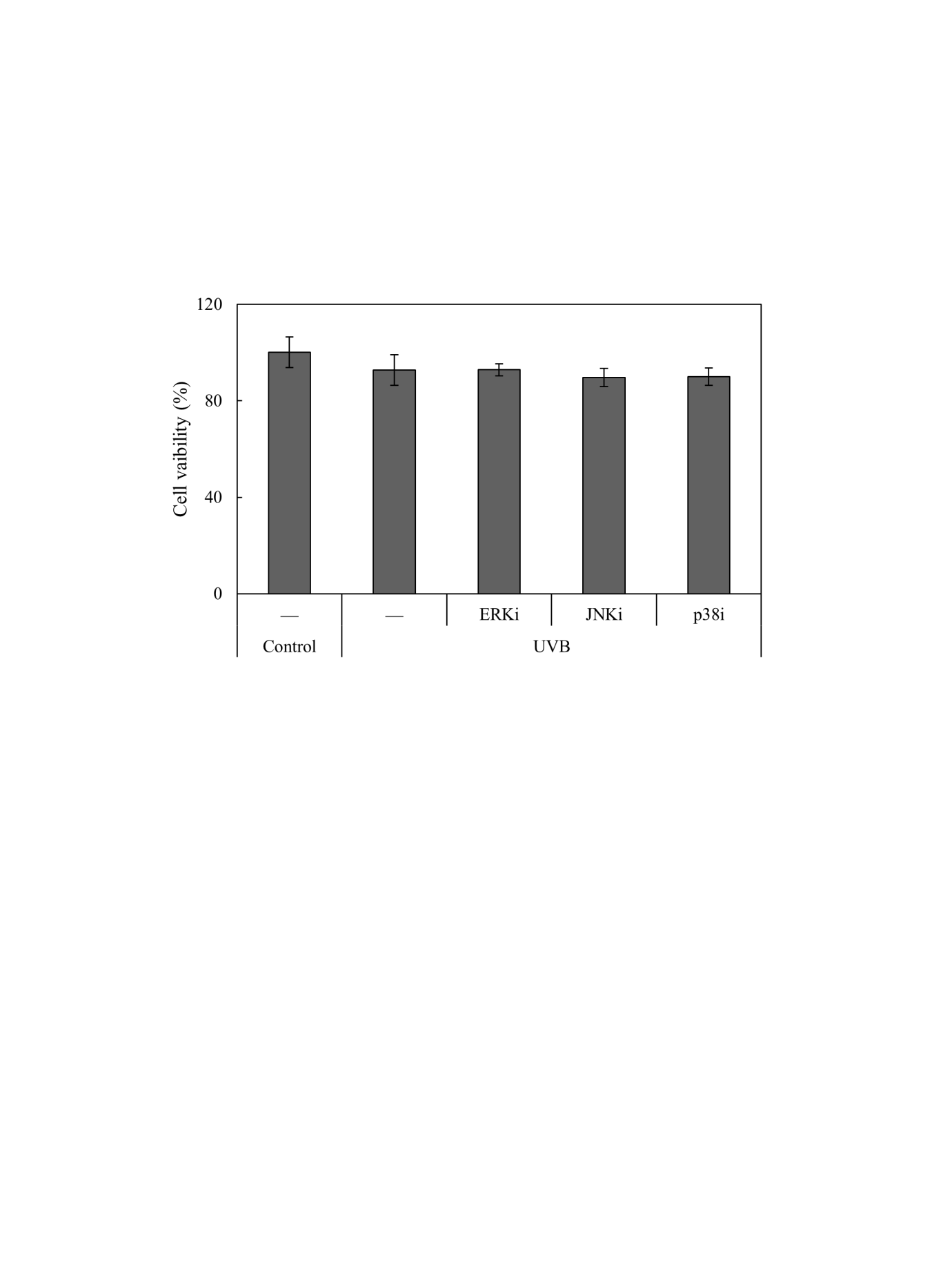
**

**Fig. S2. MAPK inhibitors do not affect cell viability after UVB exposure**

NHEKs were pretreated with the ERK (PD98059, 25 μM), p38 MAPK (SB203580, 1 μM), or JNK (SP600125, 5 μM) inhibitor for 15 min, followed by exposure to 20 mJ/cm^2^ UVB, and incubated for 24 h. Cell viability was determined by CCK-8 assay.

**
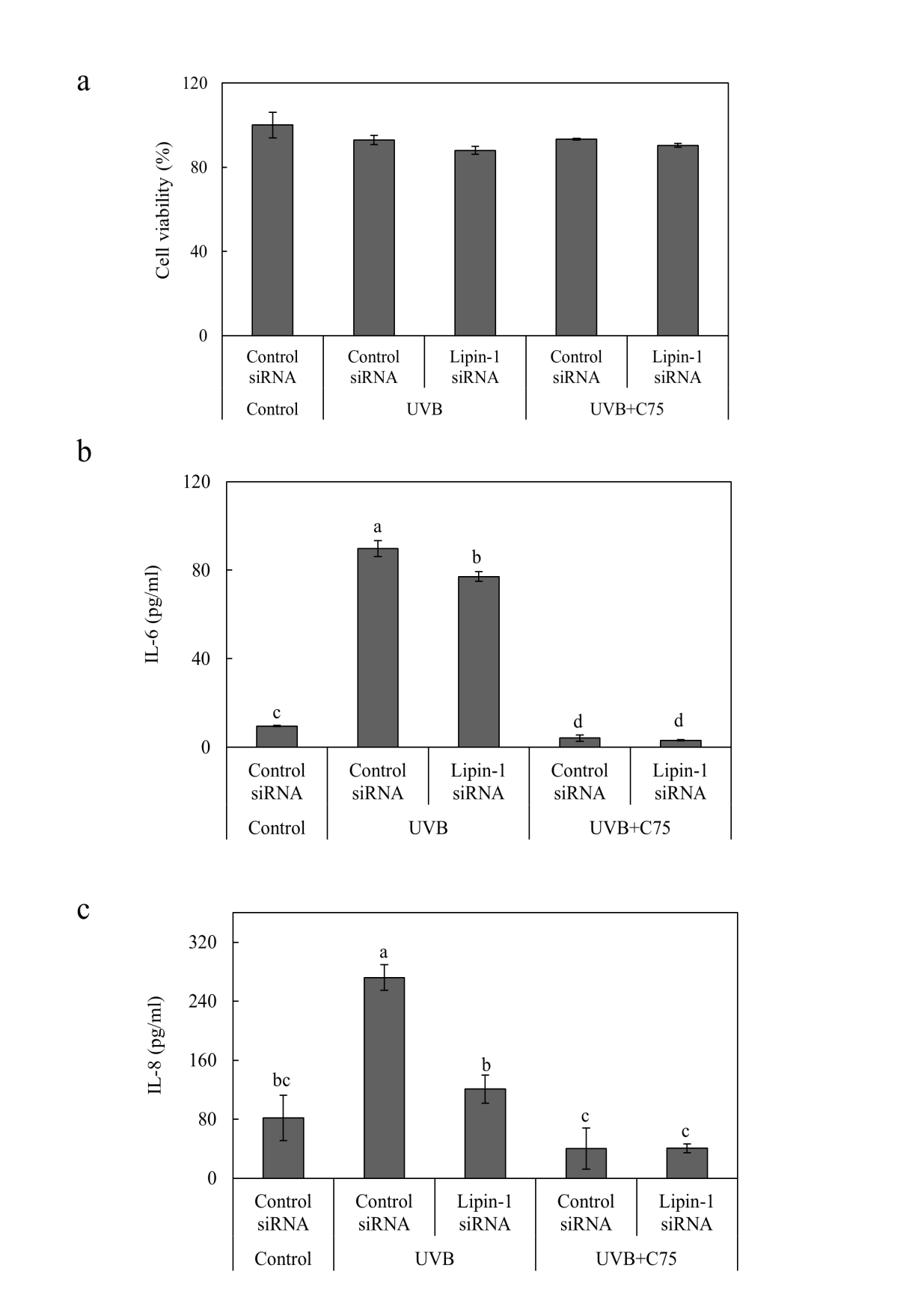
**

**Fig. S3. C75 treatment attenuates UVB-induced production of IL-6 and IL-8**

NHEKs transfected with lipin-1 siRNA or control siRNA for 24 h, exposed to 20 mJ/cm^2^ UVB radiation, and incubated in the presence of C75 (5 μM) for 24 h. (a) Cell viability was determined by CCK-8 assay. The culture media were harvested and IL-6 (b) and IL-8 (c) levels were detected via specific ELISAs. Data (mean±SD) represent three independent experiments. Means without a common letter differ; P<0.05.

**
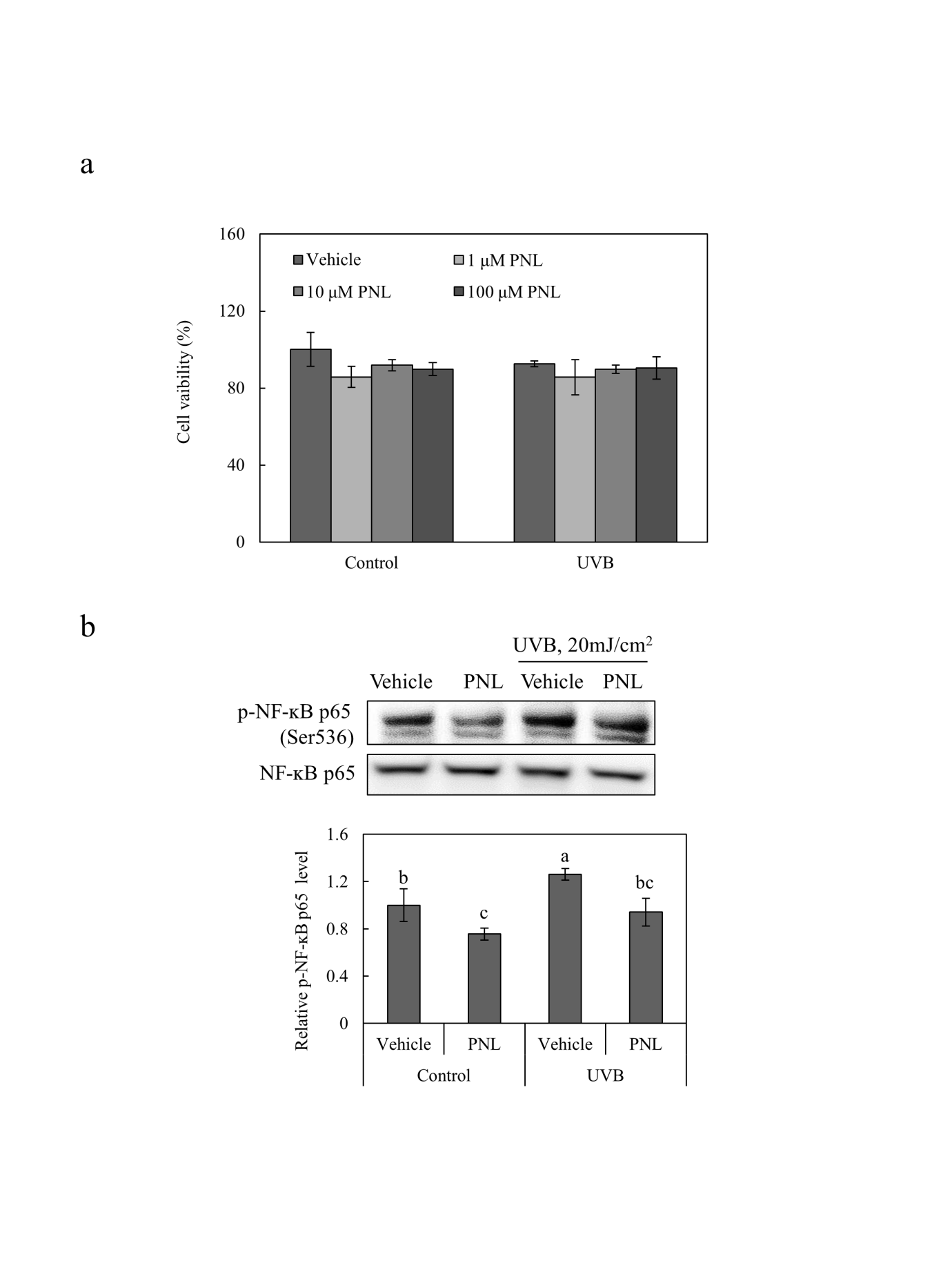
**

**Fig. S4. Propranolol attenuates** **NF-ĸB phosphorylation**

(a)NHEKs exposed with 20 mJ/cm^2^ UVB were treated with different concentrations of propranolol (PNL; 0, 1, 10, 100 μM) for 24 h. Cell viability was determined by CCK-8 assay.

(b) NHEKs exposed with 20 mJ/cm^2^ UVB were treated with 100 μM propranolol for 24 h. Immunoblot analysis was used to determine p-NF-ĸB p65 (Ser536) and NF-ĸB p65 expression levels in cell lysates. The levels of phosphorylation of NF-ĸB p65 (Ser536) were normalized to p65 NF-ĸB. Data (mean±SD) represent three independent experiments. Means without a common letter differ; P<0.05.

**
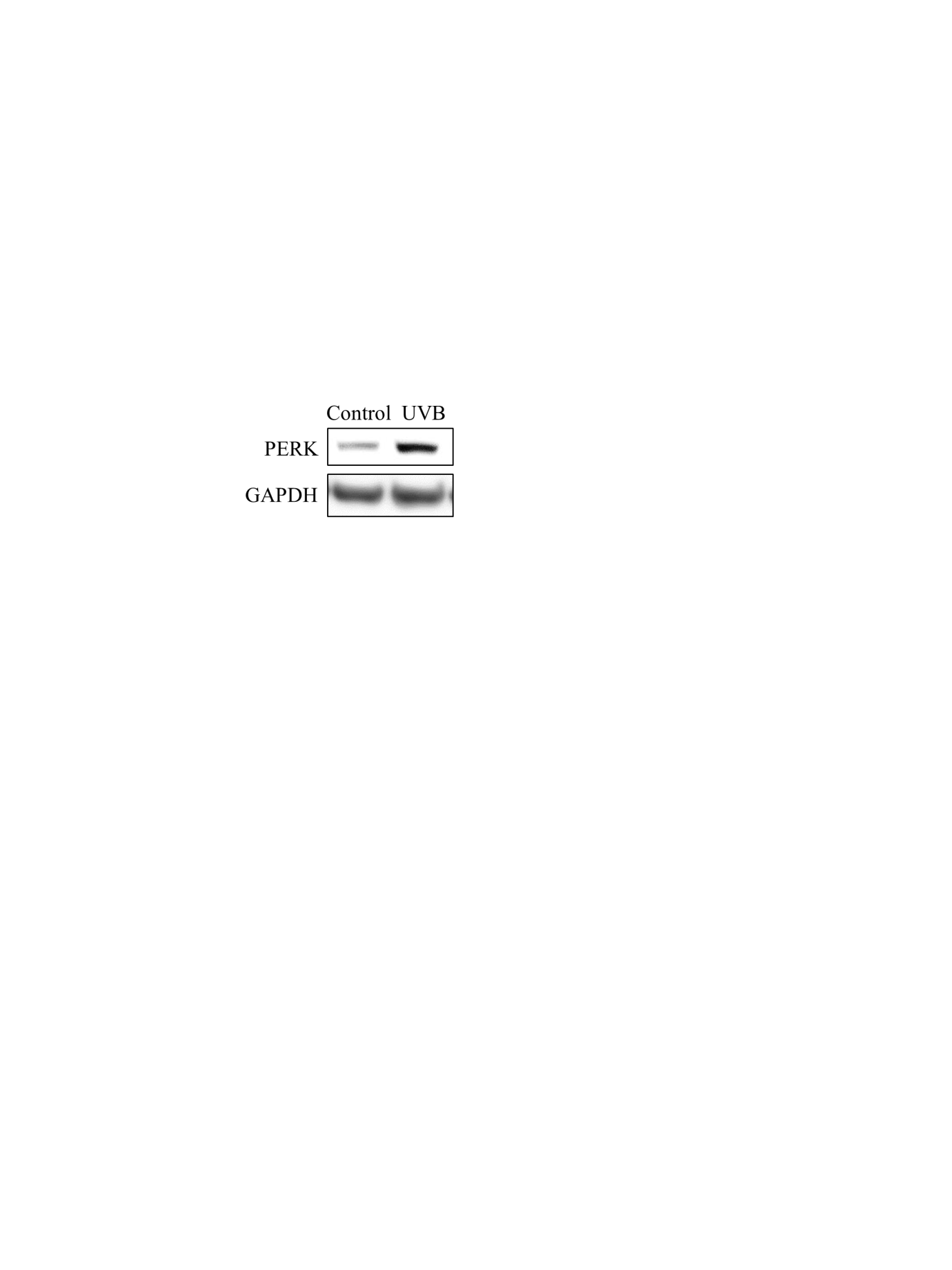
**

**Fig. S5. UVB radiation stimulates PERK expression**

NHEKs exposed with 20 mJ/cm^2^ UVB were incubated for 24h. Immunoblot analysis was used to determine PERK and GAPDH expression levels in cell lysates.
